# Effectiveness of acupuncture treatment for stroke and stroke complications: a protocol for meta-analysis and systematic review based on randomized, single-blind, controlled trials

**DOI:** 10.3389/fneur.2023.1255999

**Published:** 2023-11-02

**Authors:** Xiao-Yu Wang, Wei Liu, Huan Li, Meng-Ying Rong, Jing-Yu Li, Shao-Kang Wang, Yu-Zheng Du, Qi Zhao

**Affiliations:** ^1^Acupuncture Department, First Teaching Hospital of Tianjin University of Traditional Chinese Medicine, Tianjin, China; ^2^National Clinical Research Center for Chinese Medicine Acupuncture and Moxibustion, Tianjin, China

**Keywords:** stroke, stroke complications, acupuncture, blind design, sham acupuncture

## Abstract

**Introduction:**

The treatment and rehabilitation of stroke and its complications have become major global health issues. Acupuncture is widely used as a complementary and alternative treatment for stroke. Many clinical studies have evaluated the efficacy and safety of acupuncture, but the research results need to be more consistent. The quality of research based on previously published meta-analyzes is uneven, leading to unstable conclusions. This study aims to provide a comprehensive and systematic analysis of the efficacy of high-quality, randomized controlled trials (RCTs) based on blinded designs for treating stroke and its complications. It also aims to review the characteristics of blinded designs and the current use of sham/placebo acupuncture controls in treating stroke.

**Methods and analysis:**

This study will be conducted under the reporting guidelines for systematic reviews and meta-analyzes. Randomized controlled trials using acupuncture as the primary measure for stroke will be searched in databases such as China National Knowledge Infrastructure (CNKI), Chongqing VIP (CQVIP), Wan-fang, PubMed, Embase, Cochrane Library, and Web of Science. To evaluate high-quality research based on a blind design, if the trial evaluates the efficacy of any acupuncture intervention by including a sham/placebo acupuncture control, it will be included. The primary outcome indicator will be the ability to perform daily activities. Secondary outcome indicators include evaluating quality of life and related functions in stroke-related sequelae. We will assess the quality of evidence, reporting quality, and risk of bias for the acupuncture intervention in the literature included in this study using the GRADE system, the STRICTA 2010 checklist, and ROB2.0, respectively. RevMan 5.4 software will be used to conduct the meta-analysis, and Stata 15.0 software will be used for sensitivity analysis and publication bias testing.

**Discussion:**

By analyzing high-quality, well-designed, randomized controlled trials of acupuncture, the results of this study may contribute to a more objective and standardized evaluation of acupuncture efficacy in treating stroke and its complications.

**Systematic review registration:** PROSPERO, Identifier (CRD42023378930).

## Introduction

Stroke is the second leading cause of death and the primary cause of disability globally (1). Among neurological disorders, stroke ranks first in disability-adjusted life-years (DALY) in 21 Global Burden of Diseases (GBD) regions (2). Primary and secondary prevention, stroke units, acute treatment, and neurorehabilitation positively reduce the stroke incidence rate (1). However, the overall enhanced burden of stroke caused by the increasing prevalence of chronic stroke means that stroke is becoming a major global health problem (2, 3). According to estimates, there will be more than 70 million stroke survivors in 2030 (4). The complication of stroke mainly includes movement, sensory, epilepsy, cognitive, emotional, speech, swallowing, excretion, thromboembolism, fatigue, pain, fever, and cardiopulmonary dysfunctions. A cohort study discovered that stroke survivors often could not complete daily activities due to impairments such as hemiplegia, depression, aphasia, and cognition (5). Hence, the treatment and rehabilitation of stroke and stroke-related disabilities remains a significant problem to be solved in the coming decades.

Acupuncture is one of the oldest and most widespread treatments in traditional Chinese medicine. It has become the primary supplementary and alternative therapy recommended by the World Health Organization (WHO) due to its characteristics of being inexpensive and having less adverse effects (6). There is growing evidence to suggest that acupuncture affects the increment of blood perfusion in injured brain areas, reduction of the inflammatory reaction, stimulation of neuronal cell reorganization, and restoration of neural plasticity (7, 8). Additionally, acupuncture can facilitate the recovery of movements and emotional and cognitive function (9, 10) by widely activating various physiological pathways in the central and peripheral nervous systems (11, 12). This means that acupuncture is available for various stroke treatments and related disabilities.

The current evidence has suggested that acupuncture, a complementary and alternative therapy, has been widely recognized to treat the various clusters of symptoms occurring during stroke rehabilitation (13–16). However, the uneven quality of randomized controlled trials (RCTs) makes it difficult to evaluate acupuncture efficacy (17–19) objectively. Some studies (20–22) provide insufficient evidence to support the beneficial effect of acupuncture for stroke treatment, which might be related to methodological deficiencies in the existing acupuncture studies. Double-blind RCTs are the most rigorous way to test medical hypotheses (23, 24). However, the rigorous double-blind design of acupuncture RCTs is generally difficult to achieve because of the particularity of acupuncture operations. Therefore, most clinical studies of acupuncture adopt a single-blind design. It is essential, thus, to evaluate acupuncture treatment of stroke-related diseases in high-quality RCTs based on a blinded design. Meanwhile, sham acupuncture control, a commonly used control method for evaluating the non-specific efficacy of acupuncture, helps to meet the basic requirements of blind design (25) and promotes more definitive and meaningful conclusions in acupuncture research.

## Aims and objectives

High-quality, well-designed RCTs help improve the normative theoretical support and evidence of acupuncture treatment of stroke and its complications. This study aims to (1) estimate the efficacy of acupuncture treatment based on the blind design for stroke and stroke complications and (2) summarize the characteristics of the blind design of the current acupuncture treatment of stroke and the status quo of the control application of sham/placebo acupuncture.

## Methods and analysis

### Study registration

**Figure 1:**
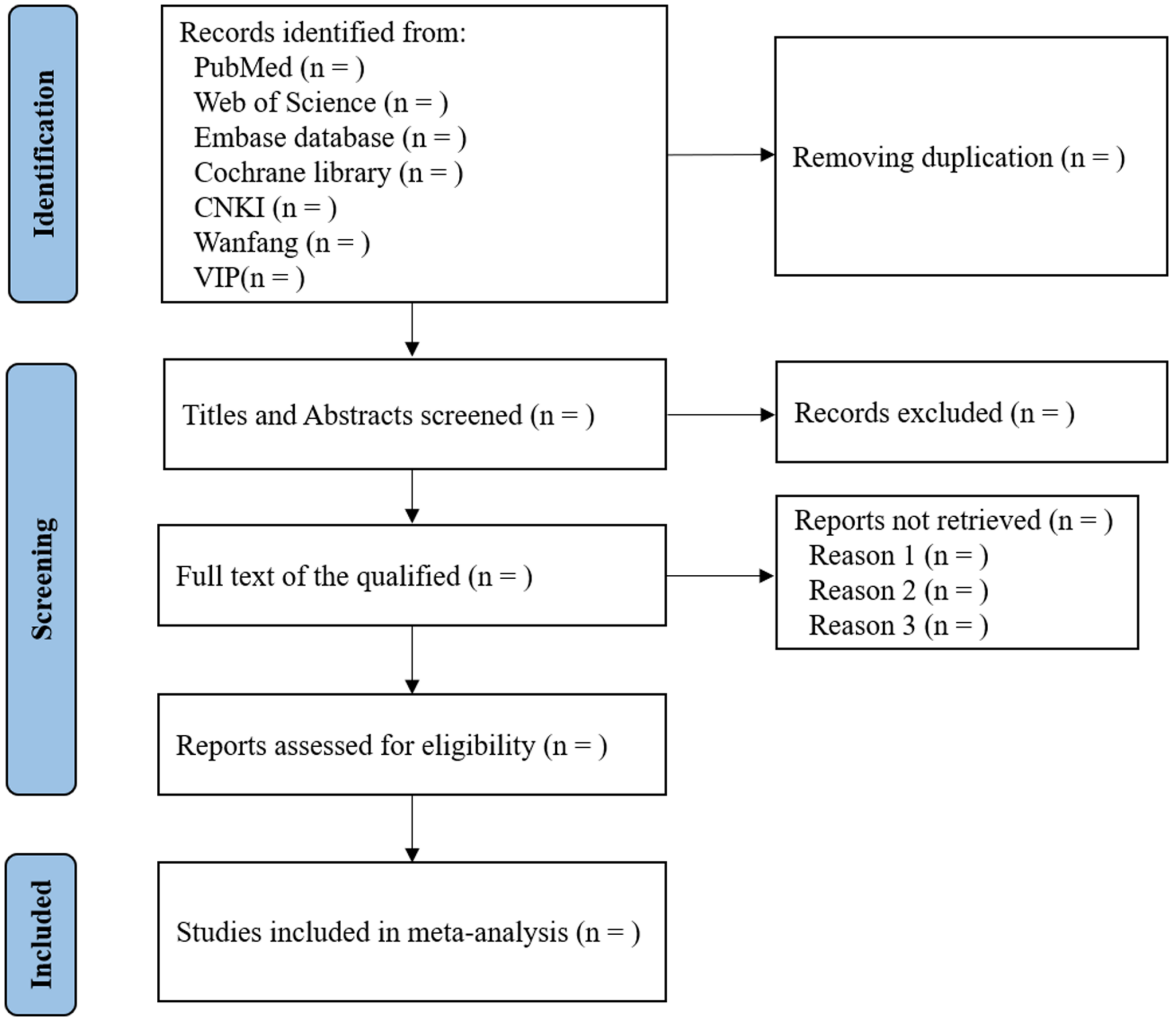
Flow diagram of the studies identified.

The protocol of this study has been registered in the Prospective Register of Systematic Reviews (CRD42023378930). The results of this systematic review and meta-analysis will be followed by the Preferred Reporting Items for Systematic Reviews and Meta-Analyzes (PRISMA) reporting guidelines. The study selection is shown in Figure 1.

### Search strategy

A comprehensive search of PubMed, Cochrane Library, Embase, Web of Science, China National Knowledge Infrastructure (CNKI), Chongqing VIP (CQVIP), and WanFang Data from 1 January 2000 to 31 August 2022, will be conducted to identify clinical trials of acupuncture in patients with stroke. Two of the authors (X-YW and WL) will perform the literature search. The association of free words and medical subject headings (MeSH) will be comprised of “stroke,” “cerebral infarction,” or “cerebral hemorrhage,” and MeSH terms “acupuncture” and “sham acupuncture,” or “placebo acupuncture,” or “non-acupoint acupuncture,” and “randomized controlled,” and not “animal.”

### Selection criteria

We will follow the PICO (population, intervention, comparison, and outcome) model to specify inclusion and exclusion criteria. We will include randomized clinical trials (RCT) in English or Chinese from 1 January 2000 to 31 August 2022.

### Types of participants

Trials involving participants of any age, sex, duration, or degree of disease with stroke (infarctional or hemorrhagic) diagnosed with CT/MRI or clinical criteria will be eligible.

### Types of interventions

Interventions in the experiment group contain traditional acupuncture and electroacupuncture. We will exclude trials in which their intervention in the experiment group involved acupoint catgut embedding therapy, acupoint injection, acupoint application, cupping, moxibustion, and bloodletting therapy.

### Types of comparators

Trials will be considered eligible if they assess the efficacy of any acupuncture intervention by including a sham/placebo acupuncture control. To ensure that the acupuncture effect can be estimated, trials with the control group receiving co-interventions not provided to the experimental group will be excluded. It will not be excluded when the trial adopts a double-blind and double-simulated design. Specifically, placebo acupuncture refers to acupuncture control methods where the needle does not penetrate the skin and only causes minor irritation to the surface of the skin. Common placebo acupunctures include blunt-headed needles and telescopic needles/devices. Sham acupuncture, a type of penetrating sham acupuncture, refers to a needle superficially placed in a region close to but not a specific therapeutic acupoint. Common sham acupunctures mainly include non-points (i.e., locations that are not known points), minimal stimulation (i.e., needling superficially), or non-meridian points (i.e., points unrelated to this disease).

### Types of outcome measures

We will include studies that used at least one of these outcome indicators: The primary outcome indicator is the daily living ability (i.e., the Barthel Index, the Activities of Daily Living, and the modified Barthel index). The secondary outcome indicators are the score of neurological defection (i.e., the National Institute of Health Stroke Scale, the modified Rankin Scale, and the Chinese Stroke Scale), life quality (i.e., the World Health Organization Quality of Life (QQL), the Stroke Specific Quality of Life Scale), and all indicators that can be used to evaluate stroke-related complications (i.e., limb function, dysphagia, depression, and cognitive or sleep disorders), and adverse events. We will include the measures used more than three times cumulatively in the meta-analysis. If the included studies used more than one indicator of the same type, we would select according to the order used in the above examples.

### Exclusion criteria

Studies lacking definite diagnoses or without full texts will be excluded. Our study will also exclude studies with issues such as missing data, incomplete data, or incorrect data expression. We will choose the most recent publication date for the article from the same research project.

## Data collection and analysis

### Study selection and data extraction

Note-express 3.7.0 will be used to manage studies. Two assessors (X-YW and WL) will independently screen the titles and abstracts of potential candidate studies for a full-text review. In case of disagreements and discrepancies between the two assessors, a third member of our team (LH) will mediate.

We will extract the following characteristics from each selected study: country, sample size, mean age, percentage of female participants, diagnosis criteria, stroke duration, precise definition and duration of intervention, pseudo-acupuncture control type, doctor qualification, blinding, time points, and content of outcome assessment. If the reported method or data is missing or unclear, the assessors will contact the corresponding author to supplement or clarify the information. Any disagreements and discrepancies will be resolved through discussion by a third reviewer (HL).

### Quality assessment

This study will use the Grades of Recommendation, Assessment, Development, and Evaluation (GRADE) approach to evaluate the treatment outcomes of acupuncture and the quality of evidence for the leading outcome indicators of included studies. The GRADE approach involves five aspects, namely, limitation, inconsistency, indirectness, imprecision, and publication bias, and the quality of evidence is divided into high, moderate, low, and very low (26). This study will use the Standards for Reporting Interventions in Controlled Trials of Acupuncture (STRICTA) 2010 checklist to assess the detailed acupuncture treatment protocol for the included studies. The STRICTA list consists of six sections, including rationality of acupuncture treatment, details of the acupuncture, treatment protocol, other interventions, therapist background, and control or controlled interventions, divided into six entries and 17 secondary items. Two assessors (X-YW and WL) will independently assess the methodologic and reporting quality for each study using the GRADE approach and the STRICTA list, respectively. A panel with the other authors will solve all discrepancies and disagreements.

### Assessment for the risk of bias

The Risk of Bias Tool (ROB2.0) will be used to assess the risk of bias of included RCTs, which consists of five dimensions of bias: randomization process, deviation from intended interventions, missing outcome data, outcome measures, and the selection of the reported results. We also assessed the risk of bias by two assessors (X-YW and WL).

### Assessment for publication bias

We will use Begg’s and Egger’s tests to assess the publication bias of these trials and form the publication bias plot. When the number of included articles exceeds 10, the funnel plot will be used to analyze whether the study has publication bias.

### Statistical analysis

All statistical analyzes will be performed using Review Manager, version 5.4, and Stata, version 15.0. We will test heterogeneity between outcomes of included studies using the I^2^ statistic. Statistically significant heterogeneity will be considered as a *p*-value of <0.10 and with I^2^ more than 50%. We will assess possible sources of heterogeneity through subgroup analysis. For the effect quantity analysis, odds ratios (ORs) will be expressed for binary outcomes (i.e., adverse effects) with 95% confidence intervals (CIs).

In contrast, for continuous outcomes (i.e., the daily living ability), the mean difference (MD) or the standardized mean difference (SMD) will be used. Given the character of the considerable clinical heterogeneity of acupuncture, we will anticipate potential heterogeneity between included studies. Therefore, the random-effects model will be used to pool effect sizes.

### Patient and public involvement

Patients will not be involved in the development of this systematic review protocol. The data for this systematic review will be collected from previously published studies.

### Ethics and dissemination

This systematic review protocol does not require ethical approval because it does not include the private information/data of the participants. This article will be published in peer-reviewed journals.

## Discussion

To the best of our knowledge, this will be the first meta-analysis exploring the effectiveness of acupuncture treatment for stroke complications based on single-blind RCTs. The number of meta-analyzes on acupuncture for stroke complications is increasing, including post-stroke motor dysfunction (27, 28), cognitive impairment (29, 30), dysphagia (31), depression (32), and insomnia (33). Nevertheless, the results of existing meta-analyzes could have been made more stable and consistent, overcoming issues of low quality and high heterogeneity, by including RCTs. The lack of blind design and results that report according to consolidated standards are significant factors of quality and heterogeneity. Moreover, whether acupuncture is merely a placebo effect has been controversial for years (34–36). Therefore, in this study, we only included single- and double-blind RCTs to provide high-quality evidence for the effectiveness of acupuncture and summarize the characteristics of sham/placebo acupuncture designs. Meanwhile, the general characteristics, such as age, gender, disease classification, and disease duration, will also be discussed in our study. This study could provide evidence to assist in decision-making among patients, caregivers, and clinicians in treating patients who have stroke complications with acupuncture and as a foundation for future studies.

Acupuncture dosage, a vital factor for heterogeneity, needs to be given more importance in meta-analyzes. Many acupuncture dosage factors, such as acupoint selection, treatment timing, manipulation technique, the background of an acupuncturist, and retention time, are essential for the acupuncture effect (37). In our study, the subgroup analysis will be conducted according to acupuncture dosage factors.

This study is also anticipated to have some limitations. First, different stroke complications and the stroke severity of the participants in included studies may lead to high potential heterogeneity and may decrease the reliability of the results. In addition, detailed information on acupuncture treatment, such as retention time and manipulation technique, may not be provided by clinical studies. The subgroup analysis on acupuncture dosage factors will be difficult to conduct with insufficient information. Finally, the number of single-blind RCTs on acupuncture treatment for stroke complications might need to be expanded to perform the analyzes.

## Ethics statement

Ethical approval was not required for the study involving humans in accordance with the local legislation and institutional requirements. Written informed consent to participate in this study was not required from the participants or the participants’ legal guardians/next of kin in accordance with the national legislation and the institutional requirements.

## Author contributions

X-YW: Conceptualization, Data curation, Formal analysis, Investigation, Software, Writing – original draft, Writing – review & editing. WL: Conceptualization, Data curation, Formal analysis, Investigation, Software, Writing – original draft, Writing – review & editing. HL: Data curation, Formal analysis, Writing – review & editing. M-YR: Data curation, Formal analysis, Writing – original draft. J-YL: Data curation, Formal analysis, Writing – original draft. S-KW: Data curation, Formal analysis, Writing – original draft. Y-ZD: Conceptualization, Writing – original draft. QZ: Conceptualization, Writing – original draft.

## References

[1] Katan M Luft A. Global Burden of Stroke. Semin Neurol. (2018) 38:208–11. doi: 10.1055/s-0038-1649503 29791947

[2] GBD 2015 Neurological Disorders Collaborator Group . Global, regional, and national burden of neurological disorders during 1990-2015: a systematic analysis for the global burden of disease study 2015. Lancet Neurol. (2017) 16:877–97. doi: 10.1016/S1474-4422(17)30299-5 28931491 PMC5641502

[3] GBD 2016 Stroke Collaborators . Global, regional, and national burden of stroke, 1990-2016: a systematic analysis for the global burden of disease study 2016. Lancet Neurol. (2019) 18:439–58. doi: 10.1016/S1474-4422(19)30034-1 30871944 PMC6494974

[4] Feigin VL Forouzanfar MH Krishnamurthi R Mensah GA Connor M Bennett DA . Global and regional burden of stroke during 1990-2010: findings from the global burden of disease study 2010. Lancet. (2014) 383:245–55. doi: 10.1016/s0140-6736(13)61953-4, PMID: 24449944 PMC4181600

[5] Kelly-Hayes M Beiser A Kase CS Scaramucci A D'Agostino RB Wolf PA. The influence of gender and age on disability following ischemic stroke: the Framingham study. J Stroke Cerebrovasc Dis. (2003) 12:119–26. doi: 10.1016/S1052-3057(03)00042-9, PMID: 17903915

[6] World Health Organization . WHO global report on traditional and complementary medicine 2019. Geneva: World Health Organization (2019).

[7] Wu P Mills E Moher D Seely D. Acupuncture in poststroke rehabilitation: a systematic review and meta-analysis of randomized trials. Stroke. (2010) 41:e171–9. doi: 10.1161/STROKEAHA.109.573576 20167912

[8] Cao BQ Tan F Zhan J Lai PH. Mechanism underlying treatment of ischemic stroke using acupuncture: transmission and regulation. Neural Regen Res. (2021) 16:944–54. doi: 10.4103/1673-5374.297061, PMID: 33229734 PMC8178780

[9] Chae Y Chang DS Lee SH Jung WM Lee IS Jackson S . Inserting needles into the body: a meta-analysis of brain activity associated with acupuncture needle stimulation. J Pain. (2013) 14:215–22. doi: 10.1016/j.jpain.2012.11.011, PMID: 23395475

[10] Zhang Y Zhang H Nierhaus T Pach D Witt CM Yi M. Default mode network as a neural substrate of acupuncture: evidence, Challenges and Strategy. Front Neurosci. (2019) 13:100. doi: 10.3389/fnins.2019.00100, PMID: 30804749 PMC6378290

[11] Sun HL Li XM. Clinical study on treatment of cerebral apoplexy with penetration needling of scalp acupoints. Chinese Acupuncture Moxibustion. (2001) 21:275–8. doi: 10.13703/j.0255-2930.2001.05.012

[12] Yang A Wu HM Tang JL Xu L Yang M Liu GJ. Acupuncture for stroke rehabilitation. Cochrane Database Syst Rev. (2016) 2016:CD004131. doi: 10.1002/14651858.CD004131.pub3, PMID: 27562656 PMC6464684

[13] NIH Consensus Conference . Acupuncture. JAMA. (1998) 280:1518–24. doi: 10.1001/jama.280.17.1518 9809733

[14] Panel O Khadilkar A Phillips K Jean N Lamothe C Milne S . Ottawa panel evidence-based clinical practice guidelines for post-stroke rehabilitation. Top Stroke Rehabil. (2006) 13:1–269. doi: 10.1310/3TKX-7XEC-2DTG-XQKH 16939981

[15] Winstein CJ Stein J Arena R Bates B Cherney LR Cramer SC . Guidelines for adult stroke rehabilitation and recovery: a guideline for healthcare professionals from the American Heart Association/American Stroke Association. Stroke. (2016) 47:e98–e169. doi: 10.1161/STR.0000000000000098, PMID: 27145936

[16] Zhang W Lou B Li J Shi W Liu X Tang J . Clinical practice guidelines of Chinese medicine rehabilitation for ischemic stroke(cerebral infarction). Rehabil Med. (2021) 31:437–47. doi: 10.3724/SP.J.1329.2021.06001

[17] Niu JF Zhao XF Hu HT Wang JJ Liu YL Lu DH. Should acupuncture, biofeedback, massage, qi gong, relaxation therapy, device-guided breathing, yoga, and tai chi be used to reduce blood pressure? Recommendations based on high-quality systematic reviews. Complement Ther Med. (2019) 42:322–31. doi: 10.1016/j.ctim.2018.10.017, PMID: 30670261

[18] Birch S Robinson N. Acupuncture as a post-stroke treatment option: a narrative review of clinical guideline recommendations. Phytomedicine. (2022) 104:154297. doi: 10.1016/j.phymed.2022.154297, PMID: 35816994

[19] Wang L Chi X Lyu J Xu Z Fu G Liu Y . An overview of the evidence to guide decision-making in acupuncture therapies for early recovery after acute ischemic stroke. Front Neurol. (2022) 13:1005819. doi: 10.3389/fneur.2022.1005819, PMID: 36313493 PMC9608668

[20] Xin Z Xue-TL D-YK. GRADE in systematic reviews of acupuncture for stroke rehabilitation: recommendations based on high-quality evidence. Sci Rep. (2015) 5:16582. doi: 10.1038/srep16582, PMID: 26560971 PMC4642304

[21] Wei JJ Yang WT Yin SB Wang C Wang Y Zheng GQ. The quality of reporting of randomized controlled trials of electroacupuncture for stroke. BMC Complement Altern Med. (2016) 16:512. doi: 10.1186/s12906-016-1497-y, PMID: 27938353 PMC5148866

[22] Xu M Li D Zhang S. Acupuncture for acute stroke. Cochrane Database Syst Rev. (2018) 2018:CD003317. doi: 10.1002/14651858.CD003317.pub3, PMID: 29607495 PMC6956658

[23] Kaptchuk TJ . The double-blind, randomized, placebo-controlled trial: gold standard or golden calf? J Clin Epidemiol. (2001) 54:541–9. doi: 10.1016/s0895-4356(00)00347-4 11377113

[24] Ferrante di Ruffano L Dinnes J Sitch AJ Hyde C Deeks JJ. Test-treatment RCTs are susceptible to bias: a review of the methodological quality of randomized trials that evaluate diagnostic tests. BMC Med Res Methodol. (2017) 17:35. doi: 10.1186/s12874-016-0287-z, PMID: 28236806 PMC5326492

[25] White AR Filshie J Cummings TM. International acupuncture research forum. Clinical trials of acupuncture: consensus recommendations for optimal treatment, sham controls and blinding. Complement Ther Med. (2001) 9:237–45. doi: 10.1054/ctim.2001.0489, PMID: 12184353

[26] Yoshida M Kinoshita Y Watanabe M Sugano K. JSGE clinical practice guidelines 2014: standards, methods, and process of developing the guidelines. J Gastroenterol. (2015) 50:4–10. doi: 10.1007/s00535-014-1016-1, PMID: 25448314

[27] Lv Q Xu G Pan Y Liu T Liu X Miao L . Effect of acupuncture on neuroplasticity of stroke patients with motor dysfunction: a Meta-analysis of fMRI studies. Neural Plast. (2021) 2021:8841720–10. doi: 10.1155/2021/8841720, PMID: 34188677 PMC8192216

[28] Liu S Zhang CS Cai Y Guo X Zhang AL Xue CC . Acupuncture for post-stroke shoulder-hand syndrome: a systematic review and Meta-analysis. Front Neurol. (2019) 10:433. doi: 10.3389/fneur.2019.00433, PMID: 31105643 PMC6498454

[29] Kuang X Fan W Hu J Wu L Yi W Lu L . Acupuncture for post-stroke cognitive impairment: a systematic review and meta-analysis. Acupunct Med. (2021) 39:577–88. doi: 10.1177/09645284211009542, PMID: 34074151

[30] Liu W Rao C Du YZ Zhang LL Yang JP. The effectiveness and safety of manual acupuncture therapy in patients with Poststroke cognitive impairment: a Meta-analysis. Neural Plast. (2020) 2020:1–15. doi: 10.1155/2020/8890521

[31] Lu Y Chen Y Huang D Li J. Efficacy of acupuncture for dysphagia after stroke: a systematic review and meta-analysis. Ann Palliat Med. (2021) 10:3410–22. doi: 10.21037/apm-21-499, PMID: 33849125

[32] Liu R Zhang K Tong QY Cui GW Ma W Shen WD. Acupuncture for post-stroke depression: a systematic review and meta-analysis. BMC Complement Med Ther. (2021) 21:109. doi: 10.1186/s12906-021-03277-3, PMID: 33794857 PMC8017746

[33] Lee SH Lim SM. Acupuncture for insomnia after stroke: a systematic review and meta-analysis. BMC Complement Altern Med. (2016) 16:228. doi: 10.1186/s12906-016-1220-z, PMID: 27430619 PMC4950252

[34] Kim TH Lee MS Lee H. Sham acupuncture is not just a placebo. J Acupunct Meridian Stud. (2022) 15:333–5. doi: 10.51507/j.jams.2022.15.6.333, PMID: 36537115

[35] McGeeney BE . Acupuncture is all placebo and here is why. Headache. (2015) 55:465–9. doi: 10.1111/head.12524 25660556

[36] Wu XK Stener-Victorin E Kuang HY Ma HL Gao JS Xie LZ . Effect of acupuncture and clomiphene in Chinese women with polycystic ovary syndrome: a randomized clinical trial. JAMA. (2017) 317:2502–14. doi: 10.1001/jama.2017.7217, PMID: 28655015 PMC5815063

[37] Fei YT Cao HJ Xia RY Chai QY Liang CH Feng YT . Methodological challenges in design and conduct of randomised controlled trials in acupuncture. BMJ. (2022) 376:e064345. doi: 10.1136/bmj-2021-064345, PMID: 35217507 PMC8868049

